# Awareness of Melanoma and Skin Self-Examination at a Tertiary Care Hospital in Pakistan: A Study of Knowledge, Attitudes, and Practices

**DOI:** 10.7759/cureus.74251

**Published:** 2024-11-22

**Authors:** Ansab Mahmood, Muhammad Saad Babar, Allah Yar Yahya Khan, Salamat Ali, Raahim A Bashir, Hafiz Zeeshan Sadiq, Amir Rasheed, Muhammad Asadullah Khalid Rana, Ammar Anjum, Haseeb Mehmood Qadri

**Affiliations:** 1 Dermatology, Imperial College London, London, GBR; 2 Surgery, Lahore General Hospital, Lahore, PAK; 3 Surgery, Akhtar Saeed Medical and Dental College, Lahore, PAK; 4 Surgery, Aziz Bhatti Shaheed Teaching Hospital, Gujrat, PAK; 5 Medicine, CMH Lahore Medical College and Institute of Dentistry, Lahore, PAK; 6 Medicine, Aziz Bhatti Shaheed Teaching Hospital, Gujrat, PAK; 7 Medicine, Aziz Fatimah Medical and Dental College, Faisalabad, PAK; 8 Medicine, Akhtar Saeed Medical and Dental College, Lahore, PAK

**Keywords:** knowledge, melanoma, pakistan, personal satisfaction, practice, self-examination, skin examination, skin neoplasms, sunscreening agents

## Abstract

Background

Melanoma, the most lethal form of skin cancer, contributes to a majority of skin cancer fatalities globally. It carries a grim prognosis with a high propensity for early metastasis. People are not aware of moles, their natural course of progression, and skin self-examination. Consistent skin examinations have demonstrated enhanced survival rates. The month of May is celebrated for melanoma awareness.

Objective

This study aims to evaluate the awareness of the general public on mole cancer and skin self-examination (SSE).

Materials and methods

This prospective, survey-based study was conducted through interviews in the outpatient department of Lahore General Hospital, Lahore, Pakistan, in May 2022. Questions based on knowledge, attitudes, and practices of melanoma and skin self-examination were asked during the survey in the native language. The collected data underwent analysis using Google Form (Google Inc., Mountain View, USA) and IBM SPSS Statistics for Windows, Version 24 (Released 2016; IBM Corp., Armonk, New York, United States). Descriptive analysis was performed by a statistician. Categorical data were analyzed to determine frequency and percentage, while continuous data were computed as mean and standard deviation.

Results

The findings reveal that a staggering 81% of the surveyed population were unaware of melanoma. A staggering 70% admitted to never having conducted self-body examinations. Interestingly, about 57% of the population had moles on their bodies, yet a significant 90% showed no concern about them. Alarmingly, 85% had never sought medical advice on this matter. On average, half of the participants believed in the protective benefits of sunscreen, umbrellas, and sunglasses. Among those interviewed, 52% recognized the severity of melanoma, while only 33% understood its potential for spreading to other organs. Additionally, 57% agreed that self-examination could facilitate early detection, and 62% believed in the efficacy of regular skin checks for improving survival rates. Impressively, 90% of the patients found the survey informative, with half expressing high satisfaction with its conduct.

Conclusion

Melanoma is a concerning diagnosis, and simple SSE can increase survival. This survey concluded that it is imperative to raise awareness regarding the risk of melanoma, the benefit of conducting self-examination, and when to seek medical help. Larger studies are needed to extract broader aspects of various factors creating barriers to awareness.

## Introduction

Melanoma is a type of skin cancer that develops from pigment-producing cells known as melanocytes present in the basal layer of the skin. It is the deadliest type of skin cancer and accounts for 4-11% of all skin malignancies. It is responsible for 75-80% of skin-cancer-related deaths worldwide [1]. New Zealand and Australia have the highest incidence and mortality rates from cutaneous melanoma in the world owing to high levels of ultraviolet radiation (UVR) [2]. In Pakistan, a total of 1795 cases of melanomas and related skin cancers among males and 1173 similar cases among females have been reported from 1994 to 2021. According to the Shaukat Khanum Memorial Cancer Hospital and Research Center's cancer registry spanning from 1994 to 2021, melanoma ranks last in the top 10 malignancies, with its frequency continuing to rise. It constitutes 2.2% of Pakistan's total cancer burden [3].

Melanoma has a very high potential for metastasis and has a poor prognosis. The malignant tumor has a propensity for early metastasis to lymph nodes, lungs, and the brain [4]. The commonly implicated risk factors in its pathogenesis include exposure to UVR, having light-colored skin, multiple atypical naevi, advanced age, sunburn in childhood, or a family history of melanomas [5]. Genetic mutations in CDKN2A and nucleotide excision repair (NER) genes have been found in hereditary melanomas. UV radiation causes damage to DNA structure that is repaired by NER pathways. Hence, disruption of these pathways leads to mutational events giving rise to melanoma [6]. Skin biopsy and histopathology are considered “the gold standard” in the diagnosis of melanoma. The histological varieties of melanoma can be superficially spreading, lentigo, nodular, mucosal, desmoplastic, or nevoid subtypes. Melan-A/MART1 and HMB45 are the most commonly used immunohistochemistry markers. Elevated Ki-67 and serum LDH levels are considered worse prognostic indicators in metastatic melanomas [6].

Surgical excision of the primary tumor along with adequate margins of surrounding tissue is the mainstay of treatment. Lymph node dissection is also carried out if the sentinel lymph node biopsy results are positive for tumor cells. Patients with distant metastatic spread also require adjuvant chemotherapy using high-dose interferon alpha-2b. Adjuvant radiotherapy of regional lymph nodes has shown an increase in local control rates. Targeted therapies, including BRAF inhibitors and immunotherapies, are the recent advances in the treatment of melanomas [7].

Various strategies attributed to the prevention of melanoma are aimed at reducing exposure to UV radiation. The usage of umbrellas and protective clothing, especially full-sleeved clothes, a hat or scarf covering the face, head, and neck, protective sunglasses, and wearing broad-spectrum sun protection factor (SPF 15 or higher) sunscreens is highly advised in bright sunlight. Skin self-examination (SSE) using ABCDE criteria (asymmetry, border irregularity, color variation, diameter > 6 mm, and evolution) is widely implicated in the early detection of melanoma. Patients who do routine SSE have a better prognosis than those who do not follow it, considering both groups have melanoma [8]. Raising awareness and educating people on preventing skin malignancies could mitigate the incidence and prevalence of malignant melanoma, with our research poised to play a pivotal role in this endeavor.

## Materials and methods

This prospective, pilot study was conducted in May 2022. The study comprised an interview-based survey of 50 patients who consented to give the interview. A questionnaire was developed on Google Docs in Urdu and English. The questionnaire had four sections based on the demographic profile of the patient and questions regarding the awareness of melanoma, related myths and facts, and the feedback of the survey. We prepared bilingual public awareness posters in English and Urdu after a detailed literature study, stating the important features of melanoma and depicting skin examination [9].

Inclusion and exclusion criteria

Patients attending the surgical outpatient department (OPD), having an age of 20 years or greater, and consenting to the survey were included. Exclusion criteria were patients referred from non-surgical departments to surgical OPD, having a Glasgow Coma Scale of less than 15, and non-native speakers.

Data collection and analysis

Each of the respondents was asked a set of questions, and the answers were recorded on Google Forms (Google Inc., Mountain View, USA). Posters were shown and read at the end of the interview, and feedback was noted. Data was transferred to IBM SPSS Statistics for Windows, Version 24 (Released 2016; IBM Corp., Armonk, New York, United States). The results were analyzed by a statistician and results were documented as graphs, charts, and tabulations.

This survey-based study was exempted from approval by the Ethical Review Board of Lahore General Hospital owing to its study design.

## Results

Of the surveyed 50 patients from the surgical OPD, 34% (17) patients belonged to the age group of 20 to 30 years. About 72% (36) of patients were male and 28% (14) were female. The majority, i.e., 52% (26) of interviewed patients, had basic literacy (elementary education).

Overall, 80% (40) of the patients had never heard of mole cancer. About 90% (45) of the patients were unbothered by the presence of moles on their body. Additionally, 14% (7) of patients had consulted a doctor for the presence of moles on their body, the most common reason being the change in the size of the mole. About 28% (14) of the patients had their whole-body examination done once in their life (Table 1). Approximately 28% (14) of the patients examined their own skin (Table 2).

**Table 1:** Awareness of melanoma among included patients, where N = 50

Questions	Yes n, %	No n, %	Maybe n, %
Have you ever heard of mole cancer?	10, 20%	40, 80%	-
Do you have mole(s) on your body?	29, 58%	21, 42%	-
Does the presence of mole(s) worry you?	5, 10%	45, 90%	-
If yes, have you ever consulted a doctor?	7, 14%	43, 86%	-
Have you ever examined your whole body?	14, 28%	36, 72%	-
Do you think the use of sunscreen has a protective role against melanoma?	24, 48%	19, 38%	7, 14%
Do you think the use of an umbrella can prevent skin cancers, including melanoma?	26, 52%	12, 24%	12, 24%
Do you think wearing protective clothing can reduce the risk of melanoma?	29, 58%	9, 18%	12, 24%
Do you think the use of sunglasses can protect from melanoma?	26, 52%	17, 34%	7, 14%

**Table 2:** Response to frequency of skin examination by participants, where N = 50

Question	Once yearly n, %	Once in six months n, %	Once monthly n, %	Once weekly n, %	Never examined n, %
Have you ever examined your whole body?	7, 14%	2, 4%	5, 10%	14, 28%	22, 44%

About 52% (26) of patients agreed upon the fact that melanoma is the deadliest of all skin cancers, and another 62% (31) agreed that the most common site of occurrence of this neoplasm is the lower limbs and upper back (Table 3). Consequently, 90% (45) of the patients had gained useful information from our study. About 50% (25) of the patients were highly satisfied, while 38% (19) of the patients were satisfied with the nature of the survey conducted. About 86% (43) of the patients were willing to transfer information to their kith and kin.

**Table 3:** Responses to myths and facts about melanoma, where N = 50

Statements	True n, %	False n, %
Melanoma is the deadliest of all skin cancers.	26, 52%	24, 48%
Ultraviolet radiations (sunlight) play a pivotal role in the pathogenesis of melanoma.	29, 57%	21, 43%
Human beings with dark skin cannot develop melanoma.	17, 34%	33, 66%
Family history plays an important role in the development of melanoma.	21, 42%	29, 58%
Melanoma can develop on mucosal surfaces, like the oral cavity.	21, 42%	29, 58%
The most common site of occurrence of melanoma is lower limbs and upper back.	31, 62%	19, 38%
Melanoma has the ability to spread to various body organs from its primary origin in skin.	17, 34%	33, 66%
Surgery is the definitive treatment of choice for melanoma.	21, 42%	29, 58%
Metastatic melanoma requires chemotherapeutic treatment.	26, 52%	24, 48%
Skin self-examination can help in early diagnosis of melanoma.	29, 58%	21, 42%
People doing regular skin examinations have better prognosis if they develop melanoma.	31, 62%	19, 38%

## Discussion

Melanoma and other skin cancers in Pakistan exhibit significant prevalence within society, as evidenced by data from one of the largest cancer treatment centers in the country. The collective cancer registry spanning from 1994 to 2021 highlighted melanoma and other skin cancers as the top 10 malignancies in the female adult population, with substantial incidence also observed among adult males [3]. Robinson et al. reported that the USA faces an escalating burden of melanoma, with an estimated doubling of new cases by 2030 [5]. This highlights the global significance of melanoma and underscores the urgency of awareness and early detection efforts worldwide.

Our analysis unveiled a strikingly low level of awareness regarding melanoma among participants, with 80% (40) indicating they had never encountered information regarding cancerous growths on moles. This finding mirrors research conducted within the Black community in the USA, where a significant percentage of participants similarly lacked awareness of melanoma [10].

Eighty-six percent of dermatologists report performing total body skin examinations at least every three years in patients with no personal or family history of skin cancer [11]. While dermatologists emphasize the importance of regular total body skin examinations, our study revealed that only 28% (14) of patients engaged in such SSE. Comparative data from Cyprus, a nation similarly abundant in sunlight, demonstrated a higher prevalence of SSE at 39.8% [12]. Additionally, our study found that 58% (29) of participants had moles on their bodies, echoing findings from Cyprus where 72.2% reported mole presence [12]. This discrepancy in self-examination rates may be attributed to factors such as inadequate public awareness campaigns, limited funding, disparities in literacy rates, and lack of institutional interest.

Moreover, a study conducted in the USA identified significant risk factors for non-melanoma skin cancer (NMSC) through multivariable analysis. These factors included age, previous history of NMSC, and skin phototype (SPT). The study also developed a simplified multivariable model and a points system for risk assessment, demonstrating high discrimination and good calibration. Furthermore, validation of the model using a separate sample of patients confirmed its effectiveness in identifying individuals at higher risk of NMSC during screening total body skin examination [11].

Alarmingly, a mere 14% (7) of participants in our study had consulted a doctor regarding suspicious moles. In contrast, a study conducted in Italy revealed that 49.5% of patients were referred to pigmented lesion clinics (PLCs) through general practitioners, suggesting a more proactive approach to skin health [13]. The discrepancy in consultation rates in the Pakistani population could stem from the lack of a robust primary care and general practitioner network. Furthermore, while our study showed only 10% (5) of our participants expressed concern over the presence of moles, Italian research indicated that 39.9% of referrals were prompted by the identification of suspicious pigmented skin lesions, either by the individual or their healthcare provider [13]. A study conducted in Switzerland reported that a significant portion of patients (44%) suspected suspicious skin lesions themselves, leading to medical consultation and subsequent diagnosis of melanoma [14]. These findings suggest a higher level of self-awareness regarding skin health in European countries.

Regarding preventive measures, 48% (24) of individuals in our survey believed in the protective role of sunscreen against melanoma, while 52% (26), 58% (29), and 58% (29) believed in the protective benefits of umbrellas, clothing, and sunglasses, respectively. In contrast, research from Greece revealed a higher prevalence of sunscreen usage, with 86% of the population employing photoprotective measures, likely attributed to extensive photoprotective education initiatives [15]. Similarly, Mueller et al. reported that in Switzerland more than 90% of participants applied sunscreen with an SPF of 30 or higher, indicating a strong adherence to photoprotective practices [14].

It is also worth mentioning that misconceptions regarding melanoma are widespread among the general public. These misconceptions can strongly influence the level of disease awareness, early detection, and overall patient outcomes. Our study has identified numerous myths that should be addressed and dealt with accordingly.

One of the crucial misconceptions identified in our study relates to the high level of ignorance concerning melanoma itself. A staggering 80% (40) of respondents stated that they had never seen any information about melanoma or cancerous moles. This lack of awareness highlights the serious issue of individuals being unable to recognize and identify a condition that could possibly prove to be fatal. This could potentially be a contributing factor toward the delays in melanoma diagnosis and treatment, as reflected in one study that reported the time between the detection of a suspicious lesion and the initiation of treatment took more than one year in 25% of the patients [12]. To dispel this myth, it is necessary to develop comprehensive educational programs aimed at increasing public understanding of melanoma, its risk factors, and the importance of early detection.

Another misconception brought to light is the perceived insignificance of the moles on the body. Among 58% (29) of the respondents in our study who reported the presence of moles, over 90% (45) of them expressed no concerns regarding them. This suggests an underappreciation of the importance of identifying mole changes and their possible association with melanoma. Any change in size, shape, or color of moles could be an important signal of melanoma diagnosis and should require clinical consultation to determine their nature promptly. It is important to address misconceptions surrounding the importance of monitoring moles and to emphasize the necessity of seeking medical attention for concerning changes to promote early detection and reduce mortality rates, as it is estimated that SSE may reduce melanoma mortality by 63% [12].

In addition, our study also highlighted the lack of awareness regarding the preventive measures of melanoma. While more than half of the participants believed in the protective benefits of umbrellas, clothing, and sunglasses, only 48% (24) believed in the protective role of sunscreen. This disparity highlights a knowledge gap in recognizing the importance of sun protection in averting melanoma. Sunscreen with a high SPF is an essential element in minimizing exposure to ultraviolet rays, which can lead to severe skin damage, and UV protection remains one of the crucial factors in primary melanoma prevention [14]. Consequently, there should be more emphasis placed on promoting comprehensive sun protection methods such as the use of sunscreen, staying in the shade or seeking shade when necessary, and wearing protective clothing items to significantly reduce the risk.

Our study also revealed a gap in the understanding of the most common site of occurrence of melanoma, with only 62% (31) of respondents believing that it primarily occurs on the lower limbs and upper back, despite them being the most common sites reported in men and women, respectively [13]. This misconception highlights the importance of educating individuals about the diverse locations where melanoma can develop, including areas not typically exposed to the sun.

One notable misconception is the belief that individuals with dark skin cannot develop melanoma, with only 34% (17) of respondents acknowledging the possibility, despite acral melanoma being fairly common in the Black community [10]. This misconception is also reflective of the overall lack of representation of Black people in the reception of health information regarding melanoma. Education campaigns must emphasize that melanoma can affect people of all skin types, and individuals with darker skin should also remain vigilant about changes in their skin [10].

Furthermore, the study identified a misconception regarding the definitive treatment of melanoma, with 42% (21) of participants falsely believing that surgery is always the preferred treatment option. While surgery is a common approach, the management of melanoma may involve a combination of treatments, including surgery, chemotherapy, immunotherapy, and targeted therapy, depending on various factors such as the stage and characteristics of the disease. Moreover, we uncovered the misconception regarding the lethality and spread of melanoma. While over half of participants recognized melanoma as the deadliest form of skin cancer, only 34% (17) believed it could metastasize to other organs from its primary origin in the skin. This shows a lack of understanding of the invasive characteristics of melanoma and its ability to metastasize to distant sites. Raising awareness of the metastatic potential of melanoma and the vital role of early detection in preventing progression is crucial to enhancing patient outcomes.

Addressing these misconceptions necessitates multifaceted approaches, including targeted educational campaigns, community outreach programs, and collaborations with healthcare providers. We can mitigate the burden of melanoma in our communities by providing accurate information about melanoma, promoting regular SSE, and encouraging early medical consultation concerning changes.

Limitations

This study has several limitations. The sample size is small, comprising only 50 patients. It exclusively focuses on outpatient cases. Additionally, the study was conducted within a limited area of Pakistan, rendering it non-representative of the entire Pakistani population. Furthermore, the questionnaire used in the study is brief and solely captures patients' personal history, lacking detailed family history information.

Clinical recommendations

At this time, it is recommended that the general population at average risk for melanoma should perform monthly self-examinations to detect any signs of melanoma development. An appropriate self-examination should be done, carefully visualizing all body parts. If signs such as new, changing/growing, and/or atypical freckles, spots, moles, and/or bumps are detected, the patient should consult their doctor immediately for further evaluation. Moreover, appropriate use of broad-spectrum sunscreen SPF ≥ 15 is recommended for all people above six months of age. Sunlight should be avoided if possible during the peak hours of 10 am to 4 pm, and if sun avoidance is not possible, protective clothing should be worn, such as long-sleeved shirts and hats.

## Conclusions

As per the findings of this study, the surveyed population exhibited alarmingly low levels of awareness regarding melanoma and its preventive measures. It also found a high distribution of moles among the study participants. Combined with the lack of skin self-examination, this highlights the importance of outreach initiatives in Pakistan. Lack of medical consultation further aggravates the potential risk of metastasis among the population. The participants showed a great extent of satisfaction, which indicates that the people are supportive of such educational engagements and initiatives. This attitude gives rise to impactful interventions to improve healthcare.

The study was primarily conducted in Lahore; therefore, it can't be generalized nationwide. However, it gives an indication of additional public health research in this area. Furthermore, it is plausible to enhance the outcomes of melanoma by encouraging regular medical checkups, raising awareness, and promoting screening strategies, thereby reducing its impacts on the population.

## Appendices

**Table 4:** Complete survey questions

Section A - Melanoma awareness		
Sr. No.	Questions	Options
1	Have you ever heard of cancer of mole?	Yes, No
2	Do you have mole(s) on your body?	Yes, No
3	Which part of your body has an abundance of moles, if any?	Yes, No
4	Does the presence of mole(s) worry you?	Yes, No
5	If yes, have you ever consulted a doctor?	Yes, No
6	If yes, what was the reason of the consultation?	-
7	Have you ever examined your whole body?	Yes, No
8	If yes, how often do you examine your skin?	Once yearly, Once in 6 months, Once monthly, Once weekly, Never examined
9	Do you think the use of sunscreen has a protective role against melanoma?	Yes, No
10	Do you think the use of an umbrella can prevent skin cancers, including melanoma?	Yes, No
11	Do you think wearing protective clothing can reduce the risk of melanoma?	Yes, No
12	Do you think the use of sunglasses can protect from melanoma?	Yes, No
Section B - Facts and myths regarding melanoma		
Sr. No.	Statements	Options
1	Melanoma is the deadliest of all skin cancers.	True/False
2	Ultraviolet radiations (sunlight) play a pivotal role in the pathogenesis of melanoma.	True/False
3	Human beings with dark skin cannot develop melanoma.	True/False
4	Family history plays an important role in the development of melanoma.	True/False
5	Melanoma can develop on mucosal surfaces, like oral cavity.	True/False
6	The most common site of occurrence of melanoma is lower limbs and upper back.	True/False
7	Melanoma has the ability to spread to various body organs from its primary origin in skin.	True/False
8	Surgery is the definitive treatment of choice for melanoma.	True/False
9	Metastatic melanoma requires chemotherapeutic treatment.	True/False
10	Skin self-examination can help in early diagnosis of melanoma.	True/False
Section C - Feedback regarding survey		
Sr. No.	Questions	Options
1	How satisfied you are with this survey?	Highly satisfied, satisfied, dissatisfied
2	Are you willing to transfer the information learned to your kith and kin?	Yes, No
